# FDG-PET predicts bone invasion and prognosis in patients with oral squamous cell carcinoma

**DOI:** 10.1038/s41598-021-94567-w

**Published:** 2021-07-26

**Authors:** Nan-Chin Lin, I-Hsien Su, Jui-Ting Hsu, Kuo-Yang Tsai, Michael Y. C. Chen

**Affiliations:** 1 School of Dentistry, China Medical University, Taichung, Taiwan; 2 Department of Oral and Maxillofacial Surgery, Changhua Christian Hospital, Changhua, Taiwan; 3 Department of Oral and Maxillofacial Surgery, Show Chwan Memorial Hospital, Changhua, Taiwan; 4 Department of Bioinformatics and Medical Engineering, Asia University, Taichung, Taiwan; 5 Division of Oral Maxillofacial Surgery, Dental Department, China Medical University Hospital, Taichung, Taiwan

**Keywords:** Cancer, Oncology

## Abstract

^18^F-fluorodeoxyglucose-positron emission tomography (FDG-PET) is widely used for tumor staging. This study sought to determine the relationship of preoperative primary tumor SUVmax (tSUVmax) with the clinicopathological features of patients with OSCC and to compare the prognostic ability of tSUVmax with that of other recurrence factors. Data of 340 patients with OSCC who were diagnosed, treated, and followed up at the Changhua Christian Hospital were retrospectively analyzed. Only patients with OSCC arising from gingiva, palate, floor of the mouth, and retromolar trigone and those who had received preoperative FDG-PET within 2 weeks before surgery were included. tSUVmax value > 9.2 was the strong predictor of bone invasion (area under the receiver operating characteristic curve, 0.844). tSUVmax value > 7.2 showed a strong association with advanced pathological T stage and recurrence factors and was associated with poor survival; tSUVmax > 7.2 showed stronger predictive power for poor disease-free survival (DFS) than pT stage and the other recurrence factors related to primary tumor. FDG-PET can be a useful supplement to contrast-enhanced computed tomography or contrast-enhanced magnetic resonance imaging for diagnosing bone invasion by OSCC. The tSUVmax value was an independent predictor of DFS in this study.

## Introduction

Oral cavity squamous cell carcinoma (OSCC) is the sixth most common cancer in the world, with a particularly high incidence in South Asia^1^. In Taiwan, it is currently the fourth and the seventh most commonly occurring malignant tumor in males and both sexes, respectively^2^. Smoking, alcohol consumption, and betel nut chewing are the major risk factors for OSCC in Taiwan^3,4^. Despite rapid advances in treatment modalities and diagnostic tools, the survival of patients with OSCC has not improved in the recent decades^5^. Therefore, identification of better prognostic tools reported in the 8th American Joint Committee on Cancer (AJCC) staging system is a key imperative^6^. Tumors adjacent to the maxilla and mandible require specific consideration of bone involvement. The extent of bone invasion affects the prognosis and the choice of treatment modalities, such as marginal or segmental mandibulectomy^1^.

^18^F-fluorodeoxyglucose-positron emission tomography (FDG-PET) is widely used across the world for tumor staging and follow-up^7–9^. FDG-PET in combination with computed tomography (CT) is well-grounded for the diagnostic work-up of patients with head and neck cancer. In the clinical applications include the detection of the occult primary metastatic cervical lymph nodes, locoregional recurrence, distant metastasis, assessment of treatment response to external beam irradiation (also in combination with chemotherapy), and surveillance for recurrence^10^. It detects the increased glucose uptake by the tumor quantified by the maximum standardized uptake value (SUVmax)^11,12^. Increased uptake of glucose by cancer cells is attributable to metabolic alterations to support malignant properties, such as upregulation of the epithelial-to-mesenchymal transition pathway (EMT)^13^. Therefore, SUVmax as a metabolic parameter may serve as a marker of aggressiveness of OSCC. Heiden et al. reported that SUVmax was associated with a variety of oncogenic alterations including EMT pathway in esophageal adenocarcinoma^14^. Yamamoto et al. also reported that non-small cell lung cancer (NSCLC) tumors that express high SUVmax are associated with a more epithelial-mesenchymal transition (EMT)-like phenotype^15^.

To the best of our knowledge, the association between the preoperative primary tumor SUVmax (tSUVmax) and bone invasion of OSCC is not well characterized in the contemporary literature. In this study, we aimed to assess the relationship of preoperative tSUVmax with the clinicopathological features, including bone invasion, tumor staging, recurrence factors, and survival outcomes, and to compare the prognostic value of tSUVmax with that of the other factors associated with recurrence.

## Material and methods

### Patients

This retrospective cohort study was approved by the Institutional Review Board and Ethics Committee of Changhua Christian Hospital, Changhua, Taiwan (IRB number is 210210). We obtained all clinical data through chart review and the cancer registry center of Changhua Christian Hospital. We confirmed that all methods were carried out in accordance with relevant guidelines and regulations. Informed consent was waived because of the retrospective nature of the study and the analysis used anonymous clinical data by the IRB of Changhua Christian Hospital, Changhua, Taiwan (IRB number is 210210). We identified 3221 patients who were diagnosed with OSCC and underwent surgery followed by systemic therapy and follow-up at the Changhua Christian Hospital between January 1, 2008, and December 31, 2019. The follow-up duration was from the date of indexing to December 31, 2019. Only patients with OSCC arising from the gingiva, palate, floor of the mouth, and retromolar trigone and those who had received preoperative FDG-PET within 2 weeks before surgery were included. The exclusion criteria were as follows: patients who did not receive treatment as per the National Comprehensive Cancer Network (NCCN) treatment guidelines, those who were lost to follow-up or for whom complete data were not available, those who were initially diagnosed with recurrence or distant metastasis, and those who did not receive treatment at the Changhua Christian Hospital. Finally, 340 patients who qualified the study selection criteria were included in the analysis.

### FDG-PET/computed tomography (CT) scan protocols

All patients who underwent FDG-PET/CT (Gemini TF16 PET scanner, Philips Healthcare, Cleveland, OH) fasted for 4–6 h prior to injection of ^18^F-fluorodeoxyglucose (FDG). Blood glucose level was checked before FDG injection to ensure that plasma glucose levels were within the range of 126–150 mg/dL. FDG-PET scan was performed 60 min after intravenous injection of 185–370 MBq (5–10 mCi) of FDG according to the body weight of patients. Non-contrast, low-dose CT scan was also performed for attenuation correction and anatomical localization; the scanning ranged from skull vertex to mid-thigh. Finally, the images were reconstructed in coronal and sagittal planes. The primary tumor SUVmax was obtained automatically by a routinely used formula described elsewhere^16^: the greatest activity response in area of interest divided by injected FDG and body weight.

### Treatment protocols

Patients enrolled in our study had undergone tumor wide excision and neck dissection according to the clinical tumor stage. Patient diagnosed with tumor superficial invaded had undergone only tumor side excision. Patients diagnosed with N0 stage had undergone selective neck dissection (levels I to III or IV), while those diagnosed with N-positive stage had undergone radical neck dissection (levels I–V). The indication for adjuvant therapy was individually determined by our interdisciplinary head and neck surgery team, which included surgeons, oncology radiologists, medical oncologist, and pathologist. Generally, postoperative radiotherapy (RT) was administered to patients with pT3 or pT4 primary tumors, N2–N3 stage nodal disease, N1 stage in level IV or V, or vascular embolism or those in whom perineural invasion was detected on pathological examination of surgical specimens. In addition, RT was optional for patients who had one positive node or perineural invasion without other adverse features. Postoperative radio-chemotherapy (CRT) was administered to patients with extracapsular nodal spread and/or positive margins. Besides, it was considered for patients with pT3 or pT4 primary tumors, N2–N3 stage nodal disease, nodal disease in level IV or V, perineural invasion, or vascular embolism. RT was administered no more than 6 weeks after operation and delivered by a linear accelerator with a dose of 60–66 Gy (1.8–2.0 Gy/fraction). If chemotherapy was indicated concurrent with RT, cisplatin (80 mg/m^2^) plus 5-fluorouracil (400–500 mg/m^2^) were administered in two cycles and repeated after 4–5 weeks.

### Clinical and pathological parameters

Patient variables included in the analysis were as follows: age at OSCC diagnosis, survival time, sex, pathological AJCC anatomic site, AJCC TNM stage, recurrence, depth of tumor invasion, grade of tumor, extranodal spread, positive lymph nodes > 2, perineural invasion, lymphovascular invasion, and close margin. In addition, history of smoking, chewing betel nuts, and alcohol consumption was also recorded. The anatomic site was subclassified into the alveolar ridge, hard palate, floor of the mouth, and retromolar trigone. Mortality data were retrieved from the cancer registry center of the Changhua Christian Hospital and from the data renewed annually by the Health Bureau of Changhua City.

### Statistical analysis

Continuous variables are presented as mean ± standard deviation, while the categorical variables are presented as frequency (percentage). Chi-squared test was used to compare the categorical variables. The effect of clinicopathological factors on disease-free survival (DFS) was examined using univariate and multivariate Cox proportional hazard model. Hazard ratios and the corresponding confidence intervals (CIs) were subsequently calculated. Estimates of the overall survival (OS) and DFS rates were calculated using Kaplan–Meier analyses. Receiver operating characteristic (ROC) curves for primary tumor SUVmax and depth of tumor invasion (DOI) were generated to determine the optimal cutoff level (using the Youden index) for predicting bone invasion and DFS; area under the ROC curves were calculated to compare the ability of primary tumor SUVmax and DOI to predict bone invasion. Comparisons of the group survival functions were conducted using the log-rank test based on the DFS. p values < 0.05 were considered indicative of statistical significance. All statistical analyses were performed using the statistical package SPSS for Windows (SPSS Inc. Released 2007. SPSS for Windows, Version 16.0. Chicago, SPSS Inc.).

## Results

**Figure 1:**
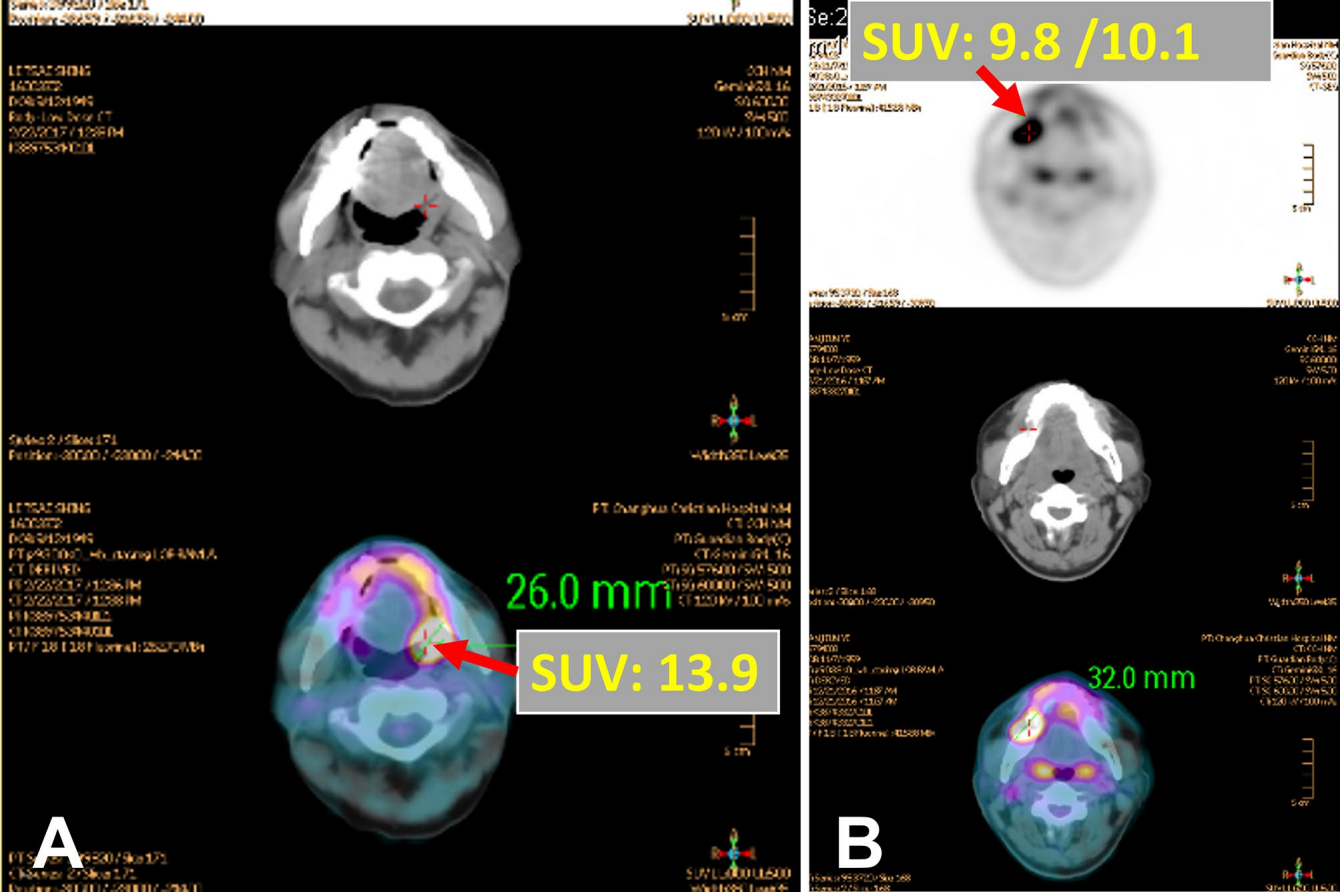
The FDG-PET image obtained from our study group. (**A**) Retromolar trigone OSCC, and the primary tumor SUVmax was 13.9. (**B**) Gingiva OSCC and primary tumor SUVmax was 10.1.

**Figure 2:**
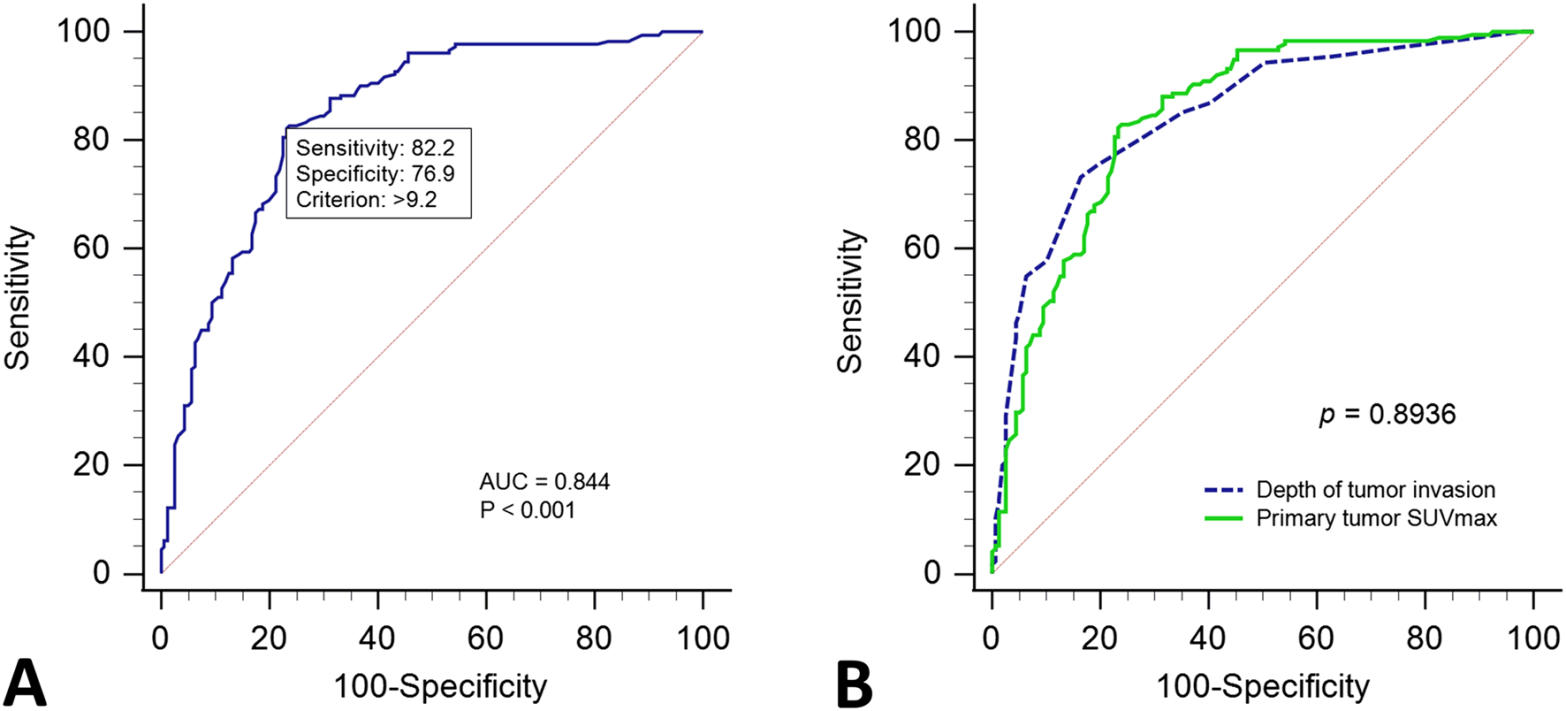
(**A**) Receiver operating characteristic (ROC) curve of primary tumor SUVmax (tSUVmax) value for predicting bone invasion of patients with pathologic positive results. The optimal cutoff point based on the Youden index is 9.2; the area under the curve (AUC) is 84.5% (95% confidence interval 0.801–0.881) (p < 0.0001). (**B**) Pairwise comparison of ROC curves of depth of tumor invasion (DOI) and tSUVmax for predicting bone invasion. The optimal DOI cutoff point for predicting bone invasion is 9 mm. AUC for DOI is 84.8% (95% confidence interval 0.805–0.885) and that for tSUVmax is 84.5% (95% confidence interval 0.801–0.881) (p = 0.8936).

A total of 340 patients diagnosed with gingiva, palate, floor of the mouth, or retromolar trigone OSCC were enrolled in our retrospective study. Figure 1 present the FDG-PET image obtained from our study group. ROC curve analyses were performed to predict the optimal cutoff level of known primary tumor SUVmax (tSUVmax) and DOI to predict bone invasion and DFS (Fig. 1). According to the Youden index^17^, the optimal cutoff tSUVmax value for predicting bone invasion was 9.2, while that for predicting DFS was 7.2. Similarly, the optimal cutoff DOI value for predicting bone invasion was 9 mm, while that for predicting DFS was 15 mm. Figure 2 shows the pairwise comparison of ROC curves of tSUVmax and DOI for predicting bone invasion; the area under the curve (AUC) for tSUVmax and DOI was 0.844 and 0.848, respectively (p = 0.8936).

The clinicopathological characteristics and the results of univariate analysis are presented in Table 1. Univariate analysis revealed a significant correlation of decreased DFS rate with advanced pathological T stage (p = 0.0216), pathological N2 stage (p < 0.0001), advanced overall stage (0.0064), tSUVmax > 7.2 (p = 0.0042), tSUVmax > 9.2 (p = 0.0051), DOI > 15 mm (p < 0.0001), bone invasion (p = 0.0029), presence of extranodal spread (p = 0.0001), perineural invasion (p = 0.0022), and lymphovascular invasion (p = 0.0499).

**Table 1:** Summary of clinicopathological features and results of univariate analysis.

	Patients (n = 340)		DFS		Univariate analysis (crude)		
	N	%	N	%	Hazard ratio	95% CI	p value
**Gender**							
Female	12	3.5	8	66.7			
Male	328	96.5	175	53.4			
**Age at diagnosis**							
≤ 40	12	3.5	9	75	0.380	0.1187–1.2136	0.102
41–50	62	62	36	58.1	0.827	0.5212–1.3122	0.420
51–60	130	130	71	54.6	1.000		
61–70	90	26.5	47	52.2	1.067	0.7202–1.5810	0.746
≥ 71	46	13.5	20	43.5	1.396	0.8794–2.2168	0.157
**Site of tumors**							
Gingiva	203	59.7	110	54.2	1.000		
Hard palate	36	10.6	16	44.4	1.349	0.8315–2.1880	0.225
Floor of mouth	46	13.5	30	65.2	0.691	0.4063–1.1750	0.172
RMT	55	16.2	27	49.1	1.145	0.7498–1.7472	0.532
**Pathological T stage**							
1	93	27.4	55	59.1	0.722	0.4953–1.0523	0.090
2	54	15.9	36	66.7	0.623	0.3765–1.0318	0.066
3	9	2.6	3	33.3	1.141	0.4998–2.6062	0.754
4	184	54.1	89	48.4	1.000		
**Pathological T stage**							
T1 + T2 stage	147	43.2	91	61.9	1.000		
T3 + T4 stage	193	56.8	92	47.7	1.467	1.0578–2.0334	0.022
**Pathological N stage**							
0	208	74	124	59.6	1.000		
1	20	7.1	15	75	0.671	0.2718–1.6540	0.386
2	49	17.4	17	34.7	2.341	1.5551–3.5225	** < 0.0001**
3	4	1.5	3	75	1.018	0.1410–7.3555	0.986
W/O ND	59						
**Pathological N stage**							
N0 + N1	225	81.1	139	61			
N2 + N3	53	18.9	20	37.7	2.316	1.5501–3.4592	** < 0.0001**
W/O ND	59						
**Stage**							
Early	124	36.5	79	63.7	1.000		
Advance	216	63.5	104	48.1	1.620	1.1453–2.2901	**0.006**
**Primary tumor SUVmax**							
SUVmax ≤ 7.2	113	33.2	73	64.6	1.000		
SUVmax > 7.2	227	66.8	110	48.5	1.692	1.1808–2.4248	**0.004**
**Primary tumor SUVmax**							
SUVmax ≤ 9.2	155	45.6	94	60.6	1.000		
SUVmax > 9.2	185	54.4	89	48.1	1.584	1.1476–2.1853	**0.005**
**Depth of tumor invasion**							
DOI ≤ 15 mm	285	83.8	166	58.2	1.000		
DOI > 15 mm	55	16.2	17	30.9	2.260	1.5645–3.2636	** < 0.0001**
**Bone invasion**							
No	160	47.1	98	61.2	1.000		
Yes	180	52.9	85	47.2	1.633	1.1829–2.2530	**0.003**
**Extranodal spread**							
No	241	85.8	144	59.8	1.000		
Yes	40	14.2	13	32.5	2.399	1.5636–3.6814	**0.0002**
W/O ND	59						
**Perineural invasion**							
No	242	71.2	142	58.7	1.000		
Yes	98	28.8	41	41.8	1.664	1.2002–2.3055	**0.002**
**Lymphovascular invasion**							
No	235	69.1	131	55.7	1.000		
Yes	105	30.9	52	49.5	1.395	1.0002–1.9450	0.050
**Positive margin involved**							
No	326	95.9	178	54.6	1.000		
Yes	14	4.1	5	35.7	1.621	0.8269–3.1783	0.160
**Grade**							
Well	35	10.3	16	45.7	1.238	0.7642–2.0061	0.386
Moderately	285	83.8	158	55.4	1.000		
Poor	20	5.9	9	45	1.531	0.8241–2.8426	0.178
**Smoking**							
No	54	15.9	24	44.4	1.000		
Yes	286	84.1	159	55.6	0.888	0.5959–1.3239	0.560
**Betel nut**							
No	103	30.3	47	45.6	1.000		
Yes	237	69.7	136	57.4	0.894	0.6426–1.2438	0.506
**Alcohol**							
No	112	32.9	52	46.4	1.000		
Yes	228	67.1	131	57.5	0.851	0.6159–1.1766	0.330

Follow-up period: from the date of diagnosis to December 30, 2019.

*W/O ND* without neck dissection, *RMT* retromolar trigone, *Early stage* AJCC stage I and II, *Advanced stage* stage III and IV.

As shown in Table 2, tSUVmax > 7.2 showed a significant association with primary tumor site (mainly at gingiva, p = 0.0008), pathological T4 stage (p < 0.0001), advanced overall stage (p < 0.0001), DOI > 15 mm (p < 0.0001), bone invasion (p < 0.0001, 71.8% cases with true bony invasion), perineural invasion (p < 0.0001), and lymphovascular invasion (p = 0.0005). As shown in Supplementary Table 1, tSUVmax > 9.2 showed the similar result to Table 1.

**Table 2:** Univariate analyses: tSUVmax ≤ 7.2 versus tSUVmax > 7.2.

	Primary tumor SUVmax				Total (n = 340)		p value
	SUVmax ≤ 7.2		SUVmax > 7.2				
	N	%	N	%	N	%	
**Gender**							
Female	2	1.8	10	4.4	12	3.5	0.2155
Male	111	98.2	217	95.6	328	96.5	
**Age at diagnosis**							
< 60	70	61.9	134	59	204	60	0.6057
> 60	43	38.1	93	41	136	40	
**Site of tumors**							
Gingiva	53	46.9	150	66.1	203	59.7	**0.0008**
Hard palate	18	15.9	18	7.9	36	10.6	
Floor of mouth	24	21.2	22	9.7	46	13.5	
RMT	18	15.9	37	16.3	55	16.2	
**Pathological T stage**							
1	73	64.6	20	8.8	93	27.4	** < 0.0001**
2	19	16.8	35	15.4	54	15.9	
3	2	1.8	7	3.1	9	2.6	
4	19	16.8	165	72.7	184	54.1	
**Pathological N stage**							
0	65	78.3	143	72.2	208	74	0.7009
1	4	4.8	16	8.1	20	7.1	
2	13	15.7	36	18.2	49	17.4	
3	1	1.2	3	1.5	4	1.4	
W/O ND	59						
**Stage**							
Early	79	69.9	45	19.8	124	36.5	** < 0.0001**
Advance	34	30.1	182	80.2	216	63.5	
**Depth of tumor invasion**							
DOI ≤ 15 mm	110	97.3	175	77.1	285	83.8	** < 0.0001**
DOI > 15 mm	3	2.7	52	22.9	55	16.2	
**Bone invasion**							
No	96	85	64	28.2	160	47.1	** < 0.0001**
Yes	17	15	163	71.8	180	52.9	
**Extranodal spread**							
No	70	87.5	171	85.1	241	85.8	0.6002
Yes	10	12.5	30	14.9	40	14.2	
W/O ND	59						
**Perineural invasion**							
No	97	85.8	145	63.9	242	71.2	** < 0.0001**
Yes	16	14.2	82	36.1	98	28.8	
**Lymphovascular invasion**							
No	92	81.4	143	63	235	69.1	**0.0005**
Yes	21	18.6	84	37	105	30.9	
**Positive margin involved**							
No	109	96.5	217	95.6	326	95.9	0.7056
Yes	4	3.5	10	4.4	14	4.1	
**Grade**							
Well	11	9.7	24	10.6	35	10.3	0.9604
Moderately	95	84.1	190	83.7	285	83.8	
Poor	7	6.2	13	5.7	20	5.9	

p value by Chi-squared test.

*W/O ND* without neck dissection, *RMT* retromolar trigone, *Early stage* AJCC stage I and II, *Advanced stage* stage III and IV.

In multivariate Cox regression analysis for DFS (Table 3), four models were conducted; these models included pathological T stage, extranodal spread, perineural invasion, lymphovascular invasion, positive margin, tSUVmax, DOI, and bony invasion. Generally, tSUVmax > 7.2, bone invasion, DOI > 15 mm, and presence of extranodal spread were significant prognostic factors for DFS.

**Table 3:** Multivariate analysis of different models for DFS.

Multivariate Cox proportional hazards regression analysis of DFS			
Risk factors	Hazard ratio	95% CI	p value
**Model 1**			
Primary tumor SUVmax (> 7.2 vs. ≤ 7.2)	1.779	1.1216–2.8221	**0.014**
Extranodal spread (Yes vs. No)	2.704	1.5020–4.8668	**0.001**
Perineural invasion (Yes vs. No)	1.110	0.7368–1.6720	0.618
Lymphovascular invasion (Yes vs. No)	0.882	0.5390–1.4446	0.619
Positive margin involved (Yes vs. No)	1.000	0.9408–1.0636	0.993
**Model 2**			
Pathological T stage (T3 + T4 vs. T1 + T2)	1.444	0.9731–2.1426	0.068
Extranodal spread (Yes vs. No)	2.559	1.4336–4.5685	**0.002**
Perineural invasion (Yes vs. No)	1.153	0.7697–1.7261	0.491
Lymphovascular invasion (Yes vs. No)	0.852	0.5219–1.3919	0.523
Positive margin involved (Yes vs. No)	0.658	0.2369–1.8285	0.422
**Model 3**			
Bone invasion (Yes vs. No)	1.797	1.2245–2.6360	**0.003**
Extranodal spread (Yes vs. No)	2.790	1.5633–4.9786	**0.001**
Perineural invasion (Yes vs. No)	1.169	0.7883–1.7344	0.437
Lymphovascular invasion (Yes vs. No)	0.784	0.4793–1.2810	0.331
Positive margin involved (Yes vs. No)	0.598	0.2141–1.6675	0.325
**Model 4**			
Depth of tumor invasion (> 15 mm vs. ≤ 15 mm)	2.211	1.4480–3.3757	**0.0002**
Extranodal spread (Yes vs. No)	2.376	1.3291–4.2459	**0.004**
Perineural invasion (Yes vs. No)	1.164	0.7768–1.7434	0.462
Lymphovascular invasion (Yes vs. No)	0.796	0.4833–1.3113	0.370
Positive margin involved (Yes vs. No)	0.822	0.2948–2.2901	0.707

Follow-up period: from date of diagnosis to December 30, 2019.

**Figure 3:**
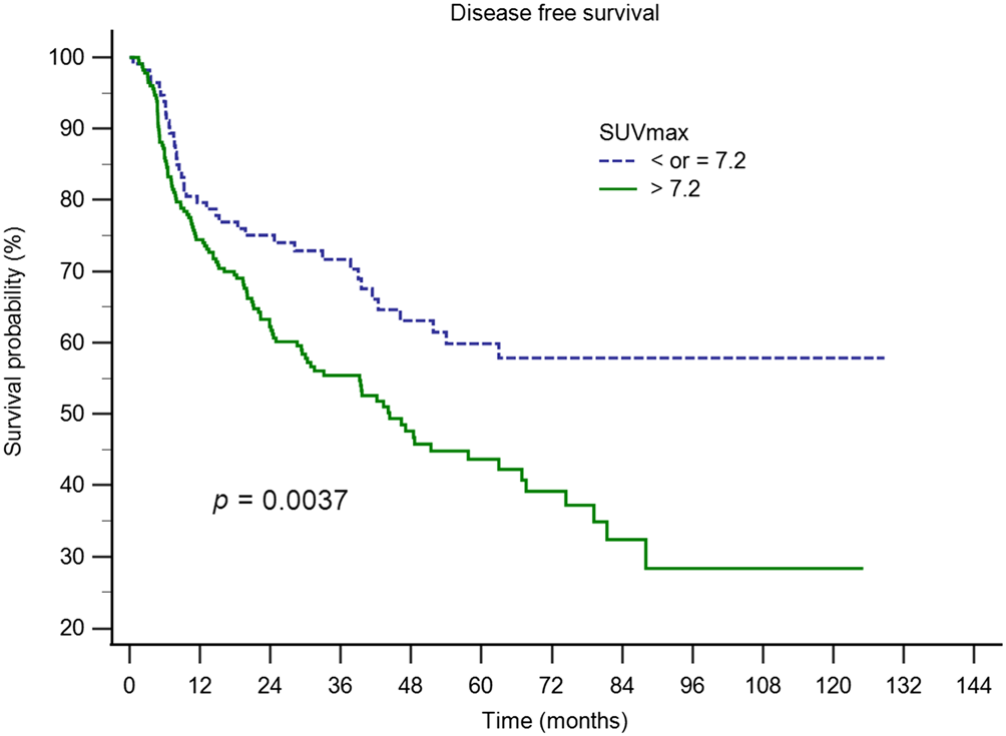
Kaplan–Meier curves for DFS of patients disaggregated into two groups using the tSUVmax cutoff value of 7.2. Results show significant difference in DFS between the two groups (p = 0.0037).

**Figure 4:**
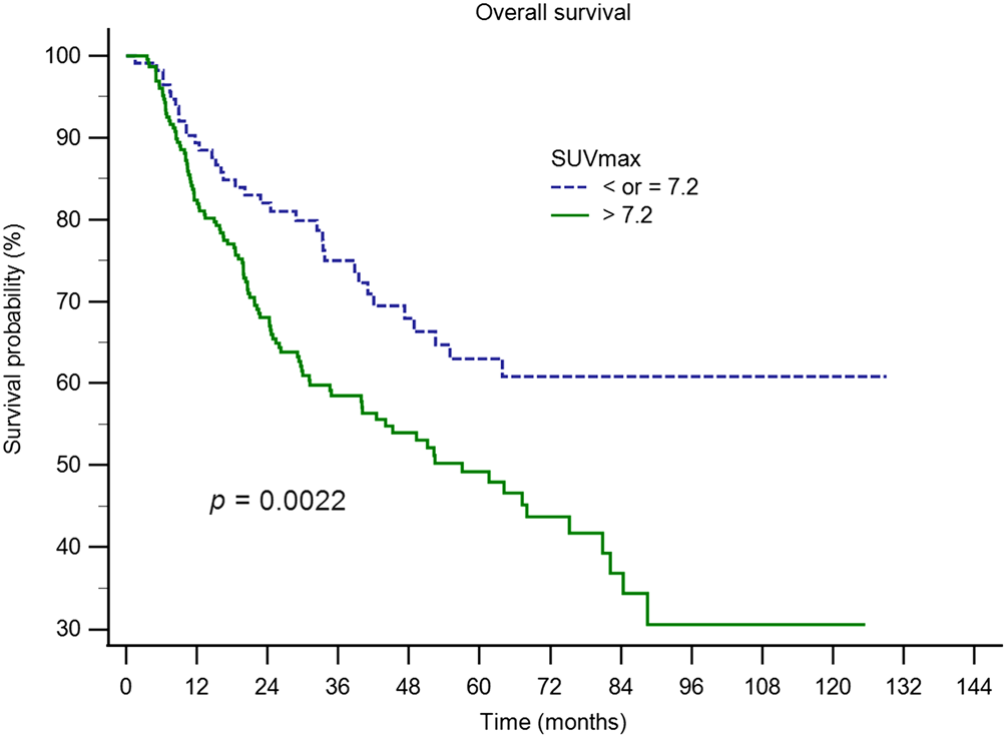
Kaplan–Meier curves for OS of patients disaggregated into two groups using the tSUVmax cutoff value of 7.2. Results show significant difference in OS between the two groups (p = 0.0022).

Figures 3 and 4 present the Kaplan–Meier curves for DFS and OS of patients disaggregated into two groups using the tSUVmax cutoff level of 7.2. These results show significant differences between the two groups with respect to DFS (p = 0.0037) and OS (p = 0.0022).

## Discussion

In this study, first, we evaluated the ability of preoperative tSUVmax to predict bone invasion and DFS. The value of tSUVmax > 9.2 was found to be the sole strong predictor of bone invasion (AUC, 0.844)^18^. Second, patients with tSUVmax > 7.2 showed a strong association with advanced pathological T stage and factors associated with recurrence (such as perineural and lymphovascular invasion). Finally, patients with tSUVmax > 7.2 showed poor DFS and OS than those with tSUVmax ≤ 7.2, while tSUVmax > 7.2 showed a stronger predictive power for poor DFS than pT stage and the other recurrence factors related to primary tumor.

Local wide excision with adequate safe margin remains the mainstay therapy for OSCC; the large defect after ablation surgery always necessitates prompt reconstructive surgery^1,19^. When jaws are involved by OSCC, the pattern of tumor ablation is directly affected by extent of tumor invasion (superficial involvement, involvement of cortical or medullary bone). Marginal mandibulectomy is typically performed for OSCC with no bone involvement or involvement of only superficial or cortical bone. Segmental mandibulectomy is reserved for patients with extensive involvement of cortical or medullary bone^20–22^. Use of free fibula flap is the most reliable and flexible approach for reconstruction of large mandibulectomy defect; therefore, meticulous preoperative planning and assessment of the extent of bony invasion by OSCC are very important^23,24^. In our study, DOI showed the same predictive ability as tSUVmax value; however, since tSUV value can be obtained preoperatively, it can inform the strategy for reconstructive surgery. Stalder et al. assessed primary tumor site SUVmax in 84 OSCC patients to predict bony invasion; they demonstrated the use of FDG-PET for preoperative diagnosis of bone invasion^25^. FDG-PET appears to be slightly better than contrast-enhanced CT (CECT) or MRI (CEMR) due to omission of their cross-sectional pattern^26^. In Stalder et al.’s study, tSUVmax values between 9.5 and 14.5 were associated with 53.6% risk of bone invasion, while tSUVmax value > 14.5 was associated with 71.4% risk of bone invasion^25^. In our analysis of 340 patients, tSUVmax > 7.2 was associated with 71.8% risk of bone invasion, while tSUVmax value > 9.2 was associated with 80% risk of bone invasion (supplementary Table 1).

In this study, tSUVmax > 7.2 was associated with more aggressive characteristics of the primary tumor, but was not related to neck metastasis or ECS. Conventional CECT or CEMR can provide information about the primary tumor and neck lymph node involvement. However, FDG-PET further renders the metabolic parameter of the tumor; SUVmax appears to be the most useful and reliable parameter as it is measured in the area with the most intensive FDG uptake. Moreover, it is easily assessed and has a high reproducibility with single voxel^27,28^. In the study of 109 nodal positive OSCC cases by Liao et al., tSUVmax was found to be an independent prognostic factor; the optimal cutoff value in their study (19.3) was higher than that in our study^29^. The discrepancy is likely attributable to two reasons: firstly, Liao et al. only enrolled nodal positive cases, i.e., patients with advanced stage OSCC, and secondly, the main survival parameter in their study was local control rate as against DFS in our study. In Suzuki et al.’s study, patients with tSUVmax > 12 had poor 3-year OS than patients who had tSUVmax below 12; in addition, tSUVmax was found to be an independent prognostic factor than clinical T and N stage for 3-year OS^30^. In contrast to 340 cases in our study, only 24 cases were enrolled; moreover, we compared the value of tSUVmax with that of the other recurrence factors. In a study of 58 patients with head and neck SCC (HNSCC) by Cacicedo et al., tSUVmax and nodal SUVmax showed no significant association with OS, DFS, or local recurrence rate^31^. Several reasons may explain the discrepancy with our study. First, in their study, they enrolled 58 HNSCC patients and only 1 OSCC patient; in addition, the treatment modalities used were completely different between each HNSCC. Second, the cases enrolled in this study belonged to an area with a high prevalence of betel nut chewing^32–36^. Finally, the small number of cases in their study may have introduced an element of bias.

Our study has several limitations that should be considered while interpreting the results. First, the dentate condition is liable to affect the results and contribute to false-positive results. Bone invasion can be more easily visualized in edentulous patients than in dentate patients^37^. Besides, FDG-PET scan is liable to be affected by inflammatory process; for example, periodontitis may cause false-positive result^37,38^. Second, the data for our study was collected from a single medical center in Taiwan. Oral cavity cancer is strongly associated with betel nut chewing, which is popular in Taiwan; therefore, our results may differ from other geographical regions^2,39^. Finally, the retrospective study design and focus on the specific anatomic sites of OSCC may have contributed to bias. Future studies should enroll all subgroups of anatomic sites of OSCC.

## Conclusions

In conclusion, FDG-PET could be a useful supplement to CECT or CEMR for diagnosis of bone invasion of OSCC. The tSUVmax value may be an independent predictor of DFS.

## Supplementary Information

Supplementary Information 1.
