# Supplementary material for: FDG-PET predicts bone invasion and prognosis in patients with oral squamous cell carcinoma
Source: Sci Rep. 2021 Jul 26;11:15153. doi: 10.1038/s41598-021-94567-w (PMC8313663; doi:10.1038/s41598-021-94567-w)
Supplement: Supplementary file 1 — Supplementary Information 1. [file 41598_2021_94567_MOESM1_ESM.docx]

**Supplementary table 1.** Summarized stratified data of age at diagnosis, anatomic tumor site, and gender.

|  |  | Primary tumor SUVmax | | | | |  |  |  |  |
| --- | --- | --- | --- | --- | --- | --- | --- | --- | --- | --- |
|  |  | SUVmax ≦ 9.2 | |  | SUVmax > 9.2 | |  | Total (n=340) | | p-value |
|  |  | N | % |  | N | % |  | N | % |  |
| Gender | Female | 2 | 1.3 |  | 10 | 5.4 |  | 12 | 3.5 | **0.0409** |
|  | Male | 153 | 98.7 |  | 175 | 94.6 |  | 328 | 96.5 |  |
| Age at diagnosis | ≦60 | 95 | 61.3 |  | 109 | 58.9 |  | 204 | 60 | 0.6571 |
|  | > 60 | 60 | 38.7 |  | 76 | 41.1 |  | 136 | 40 |  |
| Site of tumors | Gingiva | 75 | 48.4 |  | 128 | 69.2 |  | 203 | 59.7 | **0.0008** |
|  | Hard palate | 21 | 13.5 |  | 15 | 8.1 |  | 36 | 10.6 |  |
|  | Floor of mouth | 30 | 19.4 |  | 16 | 8.6 |  | 46 | 13.5 |  |
|  | RMT | 29 | 18.7 |  | 26 | 14.1 |  | 55 | 16.2 |  |
| Pathological T stage | 1 | 84 | 54.2 |  | 9 | 4.9 |  | 93 | 27.4 | **<0.0001** |
|  | 2 | 32 | 20.6 |  | 22 | 11.9 |  | 54 | 15.9 |  |
|  | 3 | 4 | 2.6 |  | 5 | 2,7 |  | 9 | 2.6 |  |
|  | 4 | 35 | 22.6 |  | 149 | 80.5 |  | 184 | 54.1 |  |
| Pathological N stage | 0 | 89 | 77.4 |  | 119 | 71.7 |  | 208 | 74 | 0.6151 |
|  | 1 | 6 | 5.2 |  | 14 | 8.4 |  | 20 | 7.1 |  |
|  | 2 | 19 | 16.5 |  | 30 | 18.1 |  | 49 | 17.4 |  |
|  | 3 | 1 | 0.9 |  | 3 | 1.8 |  | 4 | 1.4 |  |
|  | W/O ND | 59 |  |  |  |  |  |  |  |  |
| Stage | Early | 98 | 63.2 |  | 26 | 14.1 |  | 124 | 36.5 | **<0.0001** |
|  | Advanced | 57 | 36.8 |  | 159 | 85.9 |  | 216 | 63.5 |  |
| Depth of tumor invasion | DOI ≦ 15 | 148 | 95.5 |  | 137 | 74.1 |  | 285 | 83.8 | **<0.0001** |
|  | DOI > 15 | 7 | 4.5 |  | 48 | 25.9 |  | 55 | 16.2 |  |
| Bone invasion | No | 123 | 76.9 |  | 37 | 20 |  | 160 | 47.1 | **<0.0001** |
|  | Yes | 32 | 17.8 |  | 148 | 80 |  | 180 | 52.9 |  |
| Extranodal spread | No | 99 | 88.4 |  | 142 | 84 |  | 241 | 85.8 | 0.3056 |
|  | Yes | 13 | 11.6 |  | 27 | 16 |  | 40 | 14.2 |  |
|  | W/O ND | 59 |  |  |  |  |  |  |  |  |
| Perineural invasion | No | 126 | 81.3 |  | 116 | 62.7 |  | 242 | 71.2 | **0.0002** |
|  | Yes | 29 | 18.7 |  | 69 | 37.3 |  | 98 | 28.8 |  |
| Lymphovascular invasion | No | 124 | 80 |  | 111 | 60 |  | 235 | 69.1 | **0.0001** |
|  | Yes | 31 | 20 |  | 74 | 40 |  | 105 | 30.9 |  |
| Positive margin involved | No | 149 | 96.1 |  | 177 | 95.7 |  | 326 | 95.9 | 0.8343 |
|  | Yes | 6 | 3.9 |  | 8 | 4.3 |  | 14 | 4.1 |  |
| Grade | Well | 13 | 8.4 |  | 22 | 11.9 |  | 35 | 10.3 | 0.5422 |
|  | Moderately | 132 | 85.2 |  | 153 | 82.7 |  | 285 | 83.8 |  |
|  | Poor | 10 | 6.4 |  | 10 | 5.4 |  | 20 | 5.9 |  |
| p-value by Chi-square test | |  |  |  |  |  |  |  |  |  |

W/O ND: without neck dissection; RMT: retromolar trigone; Early stage: AJCC stage I and II; Advanced stage: stage III and IV
